# Hepatic and lung methotrexate-associated polymorphic lymphoproliferative disorders arising during postoperative follow-up of renal cell carcinoma: a case report

**DOI:** 10.1186/s13256-023-04288-z

**Published:** 2023-12-22

**Authors:** Yoshi Miyamoto, Chihiro Kawasoe, Kaoru Ito, Nobuyuki Oguri, Takaya Murashima, Toyoharu Kamibeppu, Takahiro Nagai, Hiroki Takamori, Toshio Kamimura, Shoichiro Mukai, Yuichiro Sato, Toshiyuki Kamoto

**Affiliations:** 1 Department of Urology, Faculty of Medicine, Miyazaki University Hospital, 5200 Kihara, Kiyotake, Miyazaki, 889-1692 Japan; 2 Department of Diagnostic Pathology, Faculty of Medicine, Miyazaki University Hospital, Miyazaki, Japan; 3 Section of Urology, Koga General Hospital, Miyazaki, Japan

**Keywords:** Methotrexate associated lymphoproliferative disorders, Renal cell carcinoma, Polymorphic lymphoproliferative disorders, Epstein–Barr virus

## Abstract

**Introduction:**

Methotrexate induces lymphoproliferative disorders on rare occasions; however, its pathogenesis remains unknown. A clinical diagnosis based on imaging studies alone is often difficult.

**Case presentation:**

A 57-year-old Japanese woman was referred to our department for the evaluation of multiple lung and hepatic nodules that developed during methotrexate treatment for rheumatoid arthritis. Since she had a history of nephrectomy for localized renal cell carcinoma, multiple lung and hepatic metastases were initially considered. However, pathological diagnosis of the lung nodules (needle biopsy) revealed methotrexate-associated polymorphic-type lymphoproliferative disorders. After methotrexate discontinuation, continuous smooth shrinkage of the lung and liver lymphoproliferative disorders was observed.

**Conclusion:**

Methotrexate-associated lymphoproliferative disorders should be considered in the event of newly appearing neoplastic lesions, even during follow-up for renal cell carcinoma, if methotrexate is being administered.

## Introduction

Methotrexate (MTX) is the most commonly used agent in the treatment of rheumatoid arthritis (RA). It was approved in Japan in 1999 and has shown high efficacy, improved quality of life, and improved life expectancy. It is currently recommended as the first-line treatment for RA. However, adverse events (AE) are not infrequent, and clinicians should be alert to gastrointestinal symptoms, liver dysfunction, infections, bone marrow suppression, interstitial pneumonia, and MTX-associated lymphoproliferative disorders (MTX-LPD). MTX-LPD is categorized into “other iatrogenic immunodeficiency-associated lymphoproliferative disorders (OIIA-LPD)” by the World Health Organization (WHO) classification (tumor of hematopoietic and lymphoid tissue) [1]. Although the etiology of MTX-LPD is unknown, its correlation with Epstein–Barr virus (EBV) infection has been indicated [1–6]. MTX-LPD commonly occurs in extranodal lesions of the skin, lung, oral cavity, pharyngeal cavities, and liver [2–7]. Here, we report a case of synchronous lung and hepatic MTX-LPD during postoperative follow-up of a patient with renal cell carcinoma (RCC).

## Case presentation

**Fig. 1:**
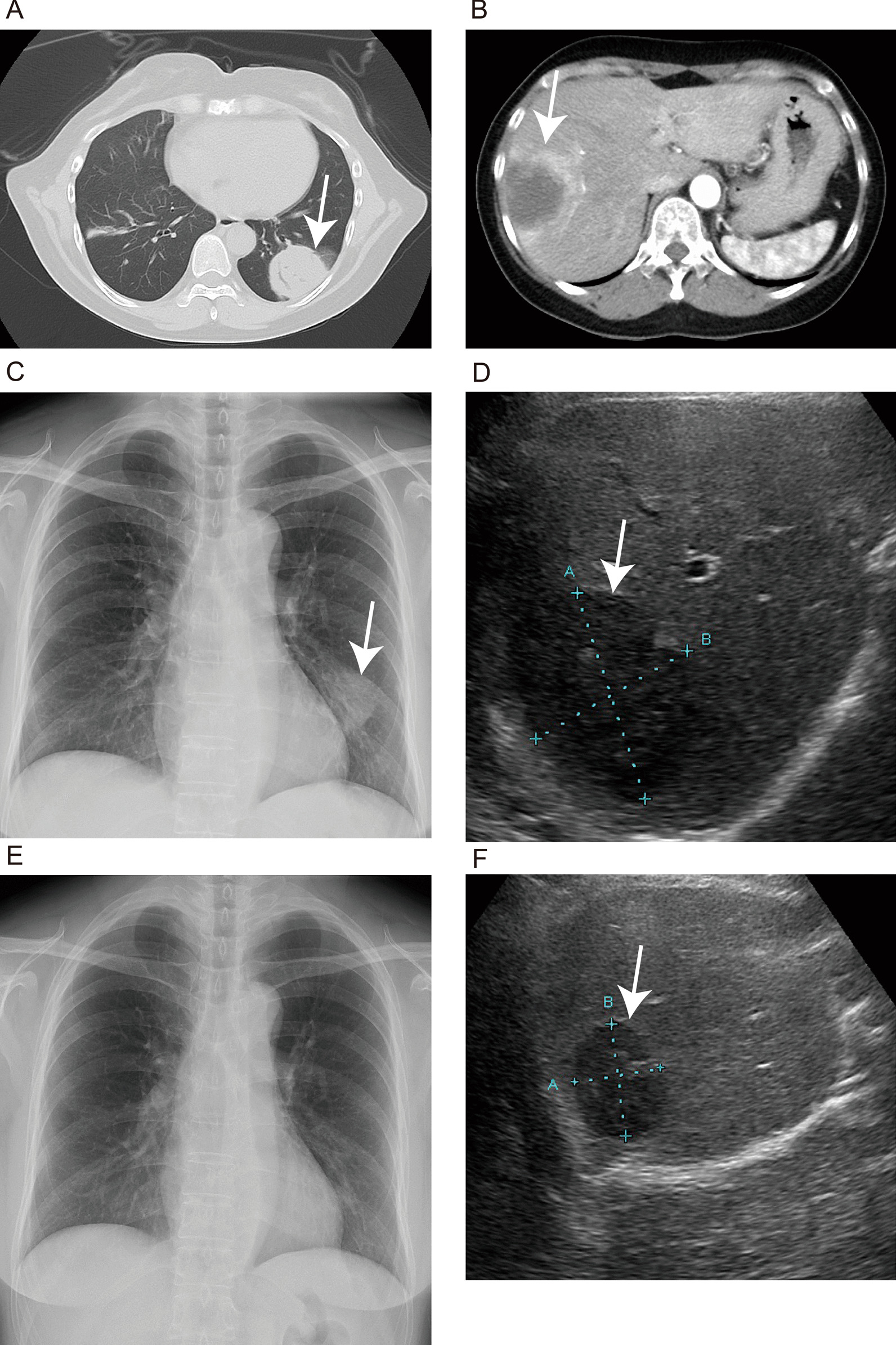
Appearance of lung and hepatic masses by computed tomography (CT), chest X-ray, and ultrasonography (US). A large lung nodule is shown on CT at the initial visit (**A**, arrow) and chest radiography at 4 weeks (**C**, arrow) and 3 months (**E**). A large hepatic nodule is shown on enhanced CT at the initial visit (**B**, arrow) and US at 4 weeks (**D**, arrow) and 3 months (**F**, arrow)

A 57-year-old Japanese woman underwent computed tomography (CT) at a familial orthopedic hospital for RA to examine a complaint of right-sided chest pain. Incidentally, CT revealed a nodule measuring 44 mm in diameter in the left lung (Fig. 1A) and a 14 mm nodule in the right lung. In addition, multiple nodules (up to 55 mm with pale ring enhancement) were observed in the liver (Fig. 1B). Because she had undergone left nephrectomy for renal cell carcinoma (RCC) 5 years prior in seeking treatment for her injured rib, multiple visceral metastases were suspected. She received annual postoperative follow-up a year prior to her visit, and no apparent evidence of recurrence was observed by thoracoabdominal CT. This prompted a referral to our department.

Details of the patient’s pathological diagnosis of the primary tumor revealed unclassified-type RCC (pT1a) without vascular and lymphatic permeation, and a Fuhrman nuclear grade of 2. When she visited our department, she had been undergoing treatment with methotrexate (8 mg/day) and prednisolone (2 mg/day) for 8 years.

**Fig. 2:**
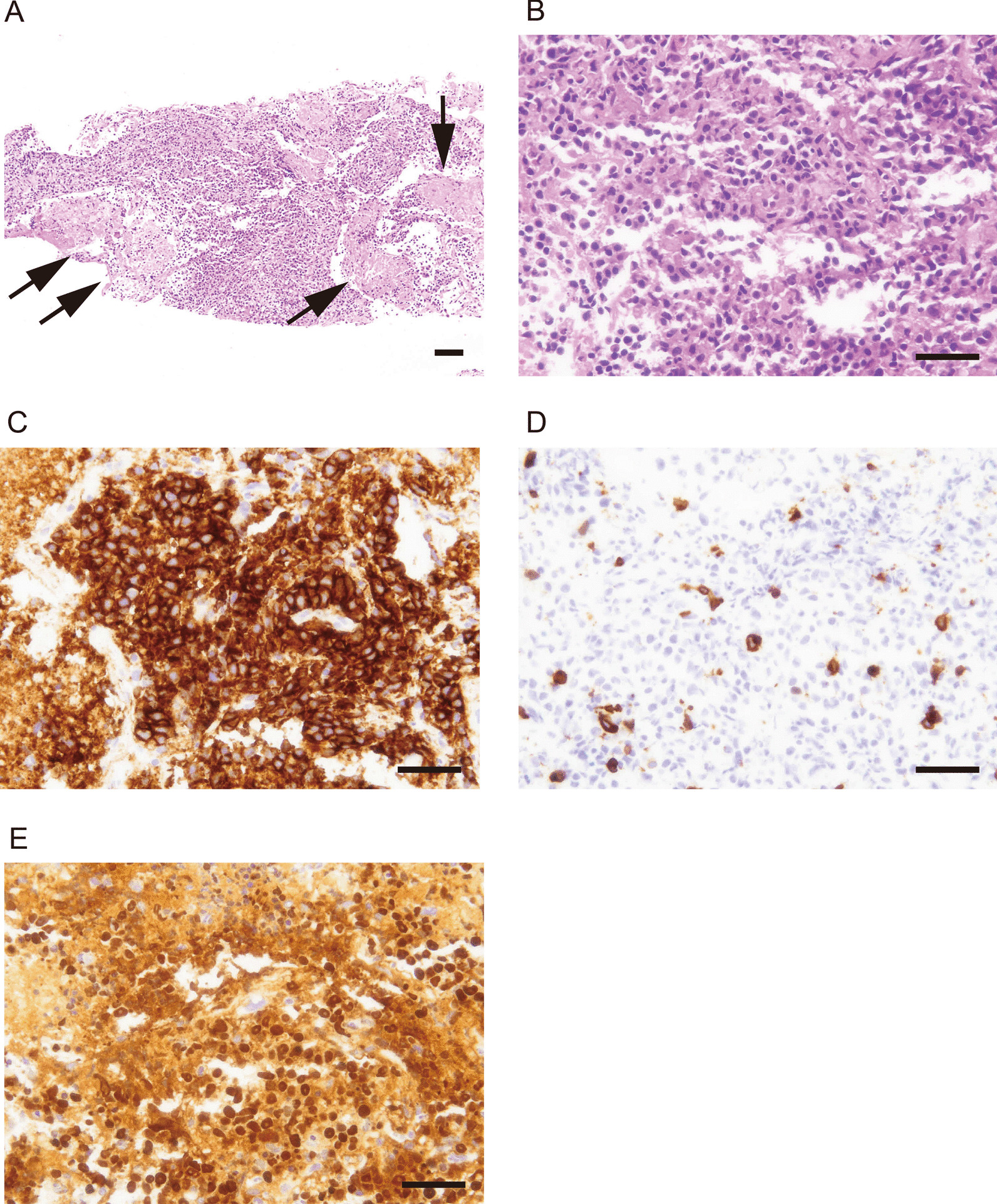
Pathological appearance of lung biopsy specimen. Variously sized lymphoid cells proliferated diffusely (**A**, **B**), and necrotic areas were observed (**A**, arrows) by hematoxylin and eosin staining. Lymphoid cells are positive for CD20 (**C**), and cCD3-positive cells are observed in the background (**D**) by immunohistochemistry. Positive EBER signals were also observed using in situ hybridization (**E**). Bars: 100 μm (**A**), 50 μm (**B**–**E**)

Blood tests showed mildly elevated aspartate aminotransferase (AST), and alanine aminotransferase (ALT): 42 IU/L and 39 IU/L, respectively, and the tumor markers for lung cancer (CEA and SCC) were within normal limits (Table 1). No apparent new mass lesion was observed by thoracoabdominal CT and the sizes of lung and liver masses were unchanged. A CT-guided needle biopsy of the lung nodule was performed to confirm the diagnosis. Pathological findings revealed diffuse proliferation of lymphoid cells (Fig. 2A, B). Necrosis and perivascular growth were also observed. Immunohistochemically, the majority of lymphoid cells were positive for CD20 and CD79a (Fig. 2C) but negative for AE1/AE3. A small amount of CD3 positive was observed (Fig. 2D). In addition, almost all lymphoid cells were positive for EBER by in situ hybridization (Fig. 2E). On the basis of these results, a diagnosis of OIIA-LPD, polymorphic LPD (poly-LPD) type was made.

**Table 1:** Summary of the laboratory data

Complete blood count		Alkaline phosphatase	157 IU/L (115–359 IU/L)
White blood cells	65 × 10^2^/μL (30–97 × 10^2^/μL)	Blood-urea-nitrogen	11.0 mg/dL (7.4–19.5 mg/dL)
Neutrophils	83.3% (36.6–79.9%)	Creatinine	0.71 mg/dL (0.5–1.2 mg/dL)
Hemoglobin	11.4 g/dL (13.1–17.6 g/dL)	Total protein	6.61 g/dL (6.4–8.3 g/dL)
Hematocrit	35.5% (38.1–50.8%)	Albumin	4.41 g/dL (3.8–5.2 g/dL)
Platelet counts	35.6 × 10^4^/μL (12.4–30.5 × 10^4^/μL)	Na	142 mEq/L (135–147 mEq/L)
Coagulation		K	4.6 mEq/L (3.4–4.8 mEq/L)
PT-INR	0.99 (0.89–1.12)	Cl	104 mEq/L (98–110 mEq/L)
APTT	28.0 seconds (23.6–31.3 seconds)	HbA1c	5.7% (4.6–6.2%)
Biochemistry		C-reactive protein	1.52 mg/dL
Total bilirubin	0.8 mg/dL (0.1–1.2 mg/dL)	Tumor marker	
Aspartate aminotransferase	42 IU/L (12–35 IU/dL)	CEA	0.8 ng/mL (0–6 ng/mL)
Alanine aminotransferase	39 IU/L (6–40 IU/L)	SCC	0.8 ng/mL (< 2.0 ng/mL)
Lactate dehydrogenase	376 IU/L (119–229 IU/L)	KL-6	510 U/mL (< 500 U/mL)
γ-glutamyl transpeptidase	71 IU/L (0–48 IU/L)		

*PT-INR* prothrombin time-international normalized ratio, *Na* sodium, *APTT* activated partial thromboplastin time, *K* potassium, *Cl* chlorine, *HbA1c* hemoglobinA1c, *CEA* carcinoembryonic antigen, *SCC* squamous cell carcinoma antigen, *KL-6* sialylated carbohydrate antigen KL-6

The methotrexate treatment was discontinued. The pulmonary mass (44 mm in size) markedly reduced to 33 mm at 4 weeks after discontinuation and disappeared at 3 months (Fig. 1C, E). After 3 months, ultrasonography revealed that the hepatic mass (55 mm in size) had reduced to 30 mm (Fig. D, F). And all other masses had also decreased in size. She is still under observation without additional treatment and has remained symptom-free for 17 months without expansion of either the lung or the hepatic LPDs.

## Discussion

In patients with a history of cancer treatment, the exclusion of metastases and primary neoplasms is necessary for visceral masses. For an accurate diagnosis, a needle biopsy was performed on the lung mass. MTX-LPD was successfully confirmed, and the subsequent treatment proceeded smoothly in our case. OIIA-LPD is classified into several histological subtypes including diffuse large B-cell lymphoma (DLBCL, 40.2–43.5%), classic Hodgkin lymphoma (CHL, 11.9–16.1%), and LPD with poly-LPD (8–15%) [1–4]. The pathological characteristics of poly-LPD include immunoblast infiltration (63%), and Hodgkin/Reed–Sternberg (HRS)-like immunoblastic cells (50%) has been reported [3]. Although capsule fibrosis, granulomatous response, and HRS-like cells are more common in poly-LPD than in DLBCL, the absence of residual follicles and abnormal karyotypes are less common [3]. Despite the limitations of the biopsy tissue, the findings in our case were generally consistent with those of poly-LPD.

Extranodal lesions have been reported in 36% of poly-LPD cases (65–70% of DLBCL, 15–43% of CHL) [2, 3]. Among the extranodal lesions of OIIA-LPDs, lung lesions were the most common (12–40%), and hepatic lesions were observed in 8–9% of cases [2–4, 7]. However, to date no case of hepatic poly-LPD has been reported. To the best of our knowledge, this is the first report of a hepatic poly-LPD.

A high EBV infection rate is also characteristic of poly-LPD (93%) compared with DLBCL (55%) and CHL (85%) [2–4]. Positive EBER staining was observed in the present case. Correlation between EBV-positivity and the regression of LPD after withdrawal of MTX has been reported; however, this phenomenon has not yet been accurately verified [2, 3, 6, 8].

As an initial treatment, discontinuation of MTX in the majority of patients with poly-LPD resulted in the resolution of lesions; however, a small number of patients (recurrence or aggressive type) received chemotherapy [3]. The response rate to MTX withdrawal (without chemotherapy) has been reported as 60–73.3% in poly-LPD, 44.3–55.6% in DLBCL, and 10.5–90% in patients with CHL [3, 4]. Cancer-related death was observed in 3% of patients with poly-LPD, 13% of patients with DLBCL, and 8% of patients with CHL [3]. As prognostic factors of poly-LPD, increased eosinophil infiltration (worse for PFS), patient age > 70 years (favorable for PFS), and extensive necrosis (favorable for PFS) have also been reported [3]. Although the patient in our case was under 70 years of age, eosinophil infiltration was not observed and necrosis was apparent. Regression of the lung and liver LPD was observed after discontinuation of MTX, and the lesions continued to shrink, suggesting relatively favorable progress.

Because this patient had a history of RCC, multiple lung and liver metastases were suspected; however, a biopsy was performed to obtain an accurate diagnosis. In previous case studies, it has been reported that accurate diagnosis of extranodal MTX-LPD is difficult using CT alone, and that biopsy is necessary [9]. Therefore, confirmation of comorbidities and treatment history, including MTX, is important. If LPD is suspected, prompt biopsy should be considered. In contrast, 18F- fluorodeoxyglucose (FDG)/positron emission tomography (PET)-CT has been reported to be useful for evaluating staging and treatment efficacy, and PET-CT is strongly recommended in conjunction with histological examination, particularly in monomorphic LPD (malignant lymphoma) [9].

## Conclusion

We report a case of synchronous lung and hepatic MTX-LPD during postoperative follow-up of a patient with RCC. An immediate biopsy of the lung nodule was performed to obtain an accurate diagnosis, and smooth regression of the LPD was observed after discontinuation of MTX. MTX-LPD should be considered for newly appearing neoplastic lesions, even during follow-up for RCC if MTX is administered to the patient.

## Data Availability

The supporting data for this report are available on request from corresponding author.

## Abbreviations

MTX: Methotrexate
RA: Rheumatoid arthritis
MTX-LPD: Methotrexate-associated lymphoproliferative disorders
OIIA-LPD: Other iatrogenic immunodeficiency-associated lymphoproliferative disorders
EBV: Epstein–Barr virus
RCC: Renal cell carcinoma
CT: Computed tomography
AST: Aspartate aminotransferase
ALT: Alanine aminotransferase
CEA: Carcinoembryonic antigen
SCC: Squamous cell carcinoma antigen
CD: Cluster of differentiation
AE1/AE3: Cytokeratin AE1/AE3
EBER: Epstein–Barr virus–encoded small RNA
Poly-LPD: Polymorphic lymphoproliferative disorders
DLBCL: Diffuse large B-cell lymphoma
CHL: Classic Hodgkin lymphoma
PFS: Progression-free survival
PET: Positron emission tomography
